# Acute Ascending Flaccid Paralysis Secondary to Multiple Trigger Factor Induced Hyperkalemia

**DOI:** 10.1155/2018/6360381

**Published:** 2018-05-29

**Authors:** K. H. D. Thilini Hemachandra, M. B. Kavinda Chandimal Dayasiri, Thamara Kannangara

**Affiliations:** ^1^Teaching Hospital Kandy, Kandy, Sri Lanka; ^2^University Paediatrics Unit, Lady Ridgeway Hospital for Children, Colombo, Sri Lanka

## Abstract

**Background:**

Acute flaccid paralysis is an uncommon, but potentially life threatening, sequel of severe hyperkalemia. Reported primary aetiologies include renal failure, Addison's disease, potassium sparing diuretics, potassium supplements, and dietary excess. Coconut water, when consumed in excess, has been reported to cause severe hyperkalemia. We report the case of acute ascending flaccid paralysis secondary to hyperkalemia induced by multiple trigger factors—king coconut water, renal failure, diabetes, metabolic acidosis, and potassium sparing diuretics.

**Case Presentation:**

A 78-year-old man presented with acute ascending type flaccid paralysis over five-hour duration and subsequently developed preterminal cardiac arrhythmias secondary to severe hyperkalemia (serum potassium: 7.02 mEq/L). He was on Losartan and Spironolactone for ischemic heart disease. Dietary history revealed excessive intake of king coconut water* (Cocos nucifera)* over past one week. Electrocardiogram returned to normal rhythm and serum potassium was 6.1 mEq/L within 2 hours of institution of emergency management for life threatening hyperkalemia. Neurological symptoms completely recovered within twenty-four hours without the need for dialysis. Electromyogram three days after the initial presentation revealed normal findings.

**Conclusions:**

The report describes a rare case of secondary hyperkalemic flaccid paralysis induced by multiple trigger factors. It is important that patients with risk factors for hyperkalemia are educated regarding avoiding excess dietary potassium. Regular follow-up of these patients is mandatory with review of medication related side effects and serum electrolytes.

## 1. Background

Hyperkalemia is an uncommon cause of reversible flaccid paralysis. While primary hyperkalemic paralysis is secondary to a defective sodium channel, a number of aetiologies have been reported as leading to secondary hyperkalemic paralysis. The primary aetiology could be one of renal failure [1], Addison's disease [2, 3], potassium sparing diuretics [4], potassium supplements, and dietary excess [5]. Coconut water, when consumed in excess, has been reported to cause severe hyperkalemia [6]. Recent reports suggest that patients with diabetes are at higher risk of developing hyperkalemia following ingestion of coconut water [7].

The objective of this report is to describe the case of a 78-year-old man with previously diagnosed diabetes mellitus, who presented with acute ascending type flaccid paralysis and subsequently developed preterminal cardiac arrhythmias following severe hyperkalemia. Increased dietary intake of king coconut* (Cocos nucifera)* water may have precipitated severe hyperkalemia in this patient in the presence of multiple other risk factors for hyperkalemia.

## 2. Case Report

A 78-year-old Sri Lankan man presented to the emergency department with acute onset upper and lower limb weakness for several-hour duration. The patient denied any limb weakness on the night prior to admission and was otherwise healthy. Upon waking up in the morning he found difficulty in getting up with weakness involving all four limbs. He was able to lift limbs but was unable to walk or dress himself. The weakness persisted and remained the same until the time of admission five hours later. Weakness was symmetrical and nonprogressive and involved all four limbs. There was no pain, numbness, or abnormal movements in limbs. Swallowing and breathing were not impaired. Urinary and faecal incontinence were absent.

Patient's past medical history consisted of type 2 diabetes, hypertension, ischemic heart disease, and systolic heart failure. Home medications included Mixtard 18 U mane and 8 U vesper, Losartan 50 mg twice daily, Nifedipine 20 mg twice daily, Spironolactone 50 mg daily, Frusemide 40 mg daily, Atorvastatin 20 mg daily, and Amiodarone 100 mg daily. Dietary history revealed that he was drinking king coconut water 2–4 servings almost every day for past one week.

He was overweight (height: 166 cm, weight: 70 kg, BMI: 25.4 kg/m^2^). Xanthelasma and arcus senilis were present. He had elevated blood pressure (140/90) with cardiac apex lying in 6th intercostal space. Examination of limbs revealed symmetrical weakness with more distal involvement (proximal 4/5, distal 3/5). Limb reflexes were not impaired except for absent bilateral ankle jerks. Impaired sensation of pain and temperature was present in a glove and stocking type distribution. Joint position sensation was diminished in both upper and lower limbs. Examination of central nervous, respiratory, and gastrointestinal systems was normal.

Investigations revealed renal dysfunction (serum creatinine: 3.66 mg/dl-, eGFR: 15 ml/min, blood urea: 20.32 mmol/l, and arterial blood HCO_3_^−^: 12.7 meq/l). He had severe hyperkalemia (serum potassium: 7.02 mmol/l) with electrocardiogram showing tall, tented T waves and sine waves. Serum sodium was 129 meq/l. Renal ultrasound showed increased cortical echogenicity and impaired corticomedullary demarcation. Noncontrast computerized tomography of brain revealed normal findings. Electromyogram did not reveal acute radiculopathy, plexopathy, or myopathy. Nerve conduction studies revealed distal segmental axonal neuropathy suggestive of diabetic polyneuropathy.

Standard ward protocol for managing hyperkalemia was followed. Patient was initially given IV 10% calcium gluconate 10 ml over 10 minutes followed by a repeat dose. Spironolactone and Losartan were immediately withheld. He was given IV 50% Dextrose 50 ml with 10 U of Soluble Insulin. Patient was nebulized with Salbutamol 5 ml over 10 minutes and it was repeated twice. The interventions showed biochemical and electrocardiographic improvement in two hours (serum potassium: 6.1 meq/l). He was commenced on calcium resonium 15 g three times daily. Limb weakness showed rapid clinical improvement within 24 hours.

## 3. Discussion

Secondary hyperkalemic paralysis is characterized by vague muscle pain and ascending muscle weakness. Clinical manifestations occur only in the presence of a potential primary aetiology. Physical examination findings include absent limb reflexes and flaccid motor paralysis. Sphincter tone and sensory function are usually not deranged. Onset is usually rapid and resolves completely following correction of hyperkalemia [8].

Hyperkalemia is well recognized complication of chronic kidney disease and the patient described in this report had previously undetected chronic kidney disease secondary to diabetic nephropathy. Hyperkalemia is clinically significant since it is associated with severe complications including fatal cardiac arrhythmias [9] and seizures [10]. Paralysis is a rare complication of hyperkalemia [11].

Coconut water, which is increasingly popular as sports drink, is reportedly a cause of fatal cardiac arrhythmia following severe hyperkalemia [12]. Eight ounces of coconut water contain 600 mg of potassium [13]. King coconut* (Cocos nucifera “king”)* is a species of coconut which is native to Sri Lanka. The recommended daily dietary intake of potassium by a person without chronic kidney disease is 4.7 g [14]. This patient had a history of short term dietary excessive intake of potassium via king coconut water and was on long term treatment with angiotensin receptor blockers and Spironolactone. Chronic kidney disease secondary to diabetic nephropathy was detected following the acute presentation. Increased dietary potassium most likely precipitated severe hyperkalemia in this patient while having other risk factors for hyperkalemia.

This presentation with ascending paralysis resembles the clinical presentation of Guillain-Barre syndrome [15]. The patient described in this report presented with rapid onset ascending type weakness over several hours without any other clinical features of Guillain-Barre syndrome. Electromyogram of this patient was normal and nerve conduction studies revealed only the long standing neuropathy. Long term hyperkalemia has been implicated as a contributing factor for neuropathy in chronic kidney disease [16]. Diabetic neuropathy is another contributing factor in this patient. Most previously reported patients had abnormal motor unit potentials in electromyograms [17] in the acute stage. This patient underwent electromyogram three days after the acute presentation since management of preterminal cardiac arrhythmias and stabilization of hyperkalemia was given priority over evaluation. Limb weakness had completely recovered by the time electromyogram was done. Electromyogram and nerve conduction velocity changes had been rapidly reversible following the correction of hyperkalemia in reported cases [18–20].

Pathophysiology and underlying genetic basis of hyperkalemic periodic paralysis are well known. There are only few case reports of secondary hyperkalemic paralysis and little is known about its underlying pathophysiology. Some reports suggest a direct influence of potassium on the muscle cell membrane/muscle fibers [15], while one report suggested a functional disturbance of the peripheral nerves [19]. Mechanism of muscle weakness and paralysis is likely due to constant and prolonged nerve membrane depolarization secondary to changes in potassium gradient and resting membrane potentials [21]. This leads to inactivation of sodium channels and impaired muscle membrane excitability. Cardiac and skeletal muscle can be adversely affected as a consequence [22].

Patients with diabetes mellitus are at a higher risk of developing hyperkalemia following ingestion of coconut water and the adverse effects are related to renal microvascular changes of diabetic nephropathy and subsequent low glomerular filtration rate. Coconut water has been found to further compromise glomerular filtration in patients with diabetic nephropathy resulting in hyperkalemia at a lower threshold [7]. Up to date there is not enough literature on recommended safe limits of daily coconut water intake for patient with comorbidities including diabetes. It is therefore important that patients with diabetes are educated regarding potential harmful effects of excessive consumption of coconut water.

The reported patient presented acute flaccid paralysis in a background of multiple trigger factors for hyperkalemia. Given the worse outcomes due to delayed treatment and complete reversibility of clinical manifestations following timely and appropriate treatment, vigilance regarding hyperkalemia is of great benefit to the patient. Further it is crucial and lifesaving that secondary hyperkalemic paralysis must be looked into while a patient is being evaluated for differential diagnosis of flaccid paralysis.

## 4. Conclusions

The reported patient had both preterminal cardiac rhythms and acute ascending flaccid paralysis following multiple trigger factor induced severe hyperkalemia. It is therefore crucial that patients with multiple risk factors for hyperkalemia are essentially educated regarding harmful effects of excess dietary potassium. Excessive consumption of coconut water can be detrimental in triggering off fatal hyperkalemia in these patients. Regular follow-up is mandatory with review of medication related side effects and serum electrolytes.

## Data Availability

The data that support the findings of this case report are available from Medical Records Department, Kandy Teaching Hospital, but restrictions apply to the availability of these data, which were used under license for the current report and so are not publicly available. Data are, however, available from the authors upon reasonable request and with permission of Medical Records Department, Kandy Teaching Hospital, Sri Lanka.
